# Reasons for Consulting a Doctor on the Internet: Web Survey of Users of an Ask the Doctor Service

**DOI:** 10.2196/jmir.5.4.e26

**Published:** 2003-10-22

**Authors:** Göran Umefjord, Göran Petersson, Katarina Hamberg

**Affiliations:** ^1^Nyland Health CenterBiskopsgatan 1SE-870 52 NylandSweden; ^2^Department of OtorhinolaryngologyLund UniversitySweden, Now at Swedish Net University AgencyHärnösandSweden; ^3^Department of Public Health and Clinical MedicineFamily MedicineUmeå UniversitySweden

**Keywords:** Internet, remote consultation, physician-patient relations, access to information, information services, anonyms and pseudonyms

## Abstract

**Background:**

In 1998 the Swedish noncommercial public health service Infomedica opened an Ask the Doctor service on its Internet portal. At no charge, anyone with Internet access can use this service to ask questions about personal health-related and disease-related matters.

**Objective:**

To study why individuals choose to consult previously-unknown doctors on the Internet.

**Methods:**

Between November 1, 2001, and January 31, 2002 a Web survey of the 3622 Ask the Doctor service users, 1036 men (29%) and 2586 (71%) women, was conducted. We excluded 186 queries from users. The results are based on quantitative and qualitative analysis of the answers to the question "Why did you choose to ask a question at Infomedica's 'Ask the Doctor' service?"

**Results:**

1223 surveys were completed (response rate 34%). Of the participants in the survey 322 (26%) were male and 901 (74%) female. As major reasons for choosing to consult previously-unknown doctors on the Internet participants indicated: convenience (52%), anonymity (36%), "doctors too busy" (21%), difficult to find time to visit a doctor (16%), difficulty to get an appointment (13%), feeling uncomfortable when seeing a doctor (9%), and not being able to afford a doctors' visit (3%). Further motives elicited through a qualitative analysis of free-text answers were: seeking a second opinion, discontent with previous doctors and a wish for a primary evaluation of a medical problem, asking embarrassing or sensitive questions, seeking information on behalf of relatives, preferring written communication, and (from responses by expatriates, travelers, and others) living far away from regular health care.

**Conclusions:**

We found that an Internet based Ask the Doctor service is primarily consulted because it is convenient, but it may also be of value for individuals with needs that regular health care services have not been able to meet.

## Introduction

Internet-based health services offer health information, including advice from health care providers, to individuals. A new service of that type is consultation with a doctor. Until now, these consultations have been mainly text-based, using communication by e-mail or by Internet servers. When the inquirer and the doctor already know each other e-mail has been the main method of communication.

Internet based Ask the Doctor services offer an opportunity for users to contact doctors they have never met. In these consultations, the inquirer may remain anonymous. We use the term *Internet doctor* for a doctor performing consultations on the Internet without any previous contact with the inquirer.

Internet consultations without a pre-existing relationship give rise to a number of questions: Why would the individual consult an Internet doctor who will have limited knowledge of the individual's medical and social background and who cannot perform a physical examination? Can this type of Internet consultation cause harm? What role will Internet consultations play in parallel with regular health care?

The experiences and benefits of Internet consultations between patients and doctors are not widely explored. In a pioneering study, conducted in 1997, Eysenbach [1] analyzed 209 questions sent by e-mail to a university dermatology hospital. The researchers found that a majority of the inquirers wanted a second opinion (while only 5% had not seen a physician before the inquiry), and that almost 1 of 5 expressed frustration with their previous patient-physician relationship. Of the inquirers, 44% asked for themselves, while 30% asked on behalf of a family member or friend. As possible reasons for why people turn to "unknown" physicians with their questions Eysenbach discusses: frustration with and lack of trust in their own physician, inadequate information received from their own physician, coping, irrational hopes, anonymity (which encourages asking embarrassing questions), and looking for information on behalf of others. Borowitz et al analyzed 1239 questions e-mailed to a unit for pediatric gastroenterology and found that the majority of the questions were sent by parents and were about the most-common intestinal disorders [2]. Legal, ethical, and clinical aspects of e-mail consultations are addressed in several papers [1,3-12]. Recently, 8 years of experiences from an Internet-based remote medical counseling project by e-mail have been described by Labiris et al [13].

With regard to consultations with Internet doctors, the experiences are primarily derived from analyzing e-mail inquiries, sometimes from situations where patients and family members write to physicians "uninvited" (unsolicited e-mail) [1]. In the present paper we studied why individuals chose to consult an "Ask the Doctor" service on the Web.

## Methods

In 1998 the Swedish noncommercial public health service Infomedica [14] opened an Ask the Doctor service on its Internet portal. At no charge, anyone with Internet access can use this service to ask questions about personal health-related and disease-related matters. The inquirer can be anonymous. Any kind of personal medical issue can be addressed without any predefined rules for the inquirer except for the mandatory input of age group and gender. Each question is answered within 7 days by experienced family doctors. Before the answer is published, it is reviewed by a coordinator. The answer is retrieved using a password. Nonpersonal or essay-type questions are rejected and responded to by a standard answer instead of being answered by an Internet doctor.

**Figure 1:**
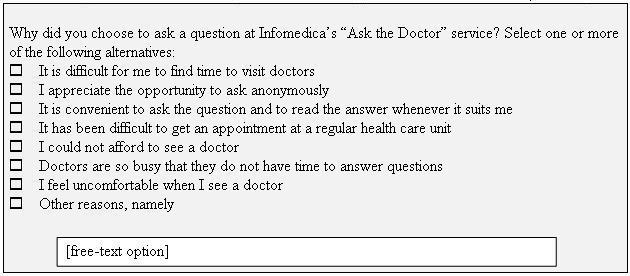
The question to be answered by the individuals using the Ask the Doctor service, with response alternatives

Between November 1, 2001, and January 31, 2002, all inquirers at Infomedica's Ask the Doctor service were invited to take part in a survey. The inquirers were informed of the survey when posing their medical question. While receiving the Internet doctor's answer on the Internet, in a separate Web-browser window the inquirer was invited to answer the question "Why did you choose to ask a question at Infomedica's 'Ask the Doctor' service?" with 7 multiple-choice alternatives and a free-text option (Figure 1). The inquirer was informed that the survey was anonymous with no possibility of the answers being traced to the respondent. The study was approved by the Umeå Clinical Research Ethics Committee, Umeå, Sweden.

In the present paper the term *inquirer* is used for an individual who posed a question to the service, and the term *participant* is used for a member of the subgroup of inquirers that also completed the survey.

### Participants

During the period of the survey a total of 3622 inquirers, 1036 men (29%) and 2586 (71%) women, used the service. Inquirers completed 1223 surveys, a response rate of 34%. It was not possible to exclude enquirers who may have posed more than 1 question to the service during the 3 months of the survey, or who may have participated in the survey more than once. There were 186 nonpersonal or essay-type questions that were rejected. Of the participants in the survey, 322 (26%) were men and 901 (74%) women. A few (n = 34) individuals who entered the study neither selected a multiple-choice alternative nor filled in the free-text box.

The inquirers indicated their age in 5-year ranges while submitting their medical question. Thus, the mean age of the inquirers could not be computed exactly, but the approximate mean age was 37 years (men: 37; women: 39) and the approximate median age of the inquirers was 35 years (men: 36; women 34). In the survey the participants defined their year of birth. The mean age for the participants was 41 years (range 8-88; men 45; women 40) and the median age was 38 years (men: 44; women: 38). Of the participants, 18 did not enter their year of birth.

### Analysis

Because the analyzed response was a combination of multiple-choice alternatives and a free-text option, both a quantitative analysis and a qualitative analysis were performed. The frequencies of the multiple-choice alternatives were computed using the software Publech version 3.0 (Ntech, Sundsvall, Sweden). The free-text answers were analyzed using a grounded theory approach [15]. After transcription the answers were read and coded for meaning and content by 2 of the researchers separately, then recoded by the 2 researchers together. Codes were discussed and sorted into categories. The reliability of the coding and categorization was discussed at a seminar by a group of researchers not involved in the study. As a result of their comments minor changes were made in the categorization.

## Results

### Multiple-choice answers

One third of the participants selected 1 multiple-choice alternative whereas two thirds selected 2 or more multiple-choice alternatives and/or gave a free-text answer (Table 1). The most frequently chosen alternative to the question "Why did you choose to ask a question at Infomedica's 'Ask the Doctor' service?"—selected by half of the participants—was convenience (Table 2). More than one third of the participants selected anonymity. Only 38 participants selected financial reasons.

**Table 1:** Distribution of selected multiple-choice and free-text alternative responses to the question in Figure 1*

**Multiple-choice Alternatives Chosen**†**, Number**	**Participants, Number**
Only multiple-choice alternative(s) chosen:	
1	380
2	252
3	136
4	47
5	13
6	1
7	0
	
Only free-text option used	177
	
Both multiple-choice alternative(s) chosen and free-text option used	
1	108
2	46
3	23
4	3
5	3
6	0
7	0
	
Neither multiple-choice alternative chosen nor free-text option used	34
	
Total	1223

^*^ The question was: "Why did you choose to ask a question at Infomedica's 'Ask the Doctor' service?"

^†^ Participants were instructed to choose 1 or more of the multiple-choice alternatives and were given the option of entering free text.

**Table 2:** Number and percentage of responders to the selected alternative responses the question in Figure 1*

**Multiple-choice Alternative Chosen**†	**Number**	**%**
It is convenient to ask the question and to read the answer whenever it suits me	640	52
I appreciate the opportunity to ask anonymously	437	36
Doctors are so busy that they do not have time to answer questions	262	21
It is difficult for me to find time to visit doctors	201	16
It has been difficult to get an appointment at a regular health care unit	163	13
I feel uncomfortable when I see a doctor	106	9
I could not afford to see a doctor	38	3
		
Subtotal	1847	
		
Other reasons, entered in [free-text option]	360	29
		
Total	2207	

^*^ The question was: "Why did you choose to ask a question at Infomedica's 'Ask the Doctor' service?"

^†^ Participants were instructed to choose 1 or more of the multiple-choice alternatives and were given the option of entering free text. There were 1223 participants.

### Free-text answers

More than one fourth, 360 participants, chose to use the free-text box. In the qualitative analysis these answers were coded and sorted into the following 6 categories, listed in order of decreasing prevalence. Some of the answers included more than one reason and were sorted into more than one category.

#### Second Opinion (in 110 of 360 free-text answers, 31%)

A wish for a second opinion was the most-common reason among the free-text answers, expressed by more than one fifth of the participants. Many just wrote the words "second opinion" while others gave a more detailed explanation, for example, "It is good to ask someone else. Everyone does not have the same opinion." Several of the free-text answers disclosed that family members wanted a second opinion on behalf of relatives.

#### Discontent With Previous Doctors (89/360, 25%)

Almost as frequent as wanting a second opinion were answers expressing discontent with health care previously received and in particular discontent with doctors. Many participants complained that their doctor "did not know the answer" or that doctors had "given contradictory answers." Others claimed that their doctor "did not care," "did not listen," "was short of time," "was nonchalant," "was negligent," or "did not rack his brain with the problem." Some participants complained that the doctor was "hard to understand" because of language difficulties.

#### Primary Evaluation of a Medical Problem (53/360, 15%)

In this category, some respondents wanted to know if it was necessary to visit a physician at all. Some participants were uncertain if their question was severe enough to bother a doctor at his/her clinic, for example, "feeling foolish, it might not be serious." Others wanted deeper knowledge of body functions claiming that this was not often accomplished while seeing a physician. In a few cases the reason for asking was the explicit wish of remaining autonomous and taking care of the health issues oneself, for example, "wanted to check if I could do anything myself without seeing a doctor." Some wanted to get further knowledge before an appointment, for example, "I want to prepare myself before visiting my doctor. Get knowledge. Get alternative points of view."

#### Convenience, Distance, and Time (49/360, 14%)

Although it was a multiple-choice alternative, some participants also used the free-text option to express their satisfaction with the possibility of using a computer to pose their question whenever it suited them, for example, "This is faster and it is more convenient to use the computer." Some lived in rural areas with few doctors. At least 10 of the participants were Swedes living abroad wanting to consult a doctor in their native language. Discontent with access to regular health care was also a frequent complaint. Some had been offered an appointment with a doctor in the distant future but did not want to wait that long.

#### Embarrassing Concerns and Worries (16/360, 4%)

A few participants expressed their appreciation for the option of getting answers to embarrassing questions, for example, "I feel that my problems are a bit awkward." Others stated worry as the main reason for asking, "still worried although I have already seen a doctor" and in a couple of cases also presented himself/herself as a hypochondriac.

#### Preference for Written Communication (15/360, 4%)

A few participants stated that both the question and the answer could be better formulated when communicating in writing, for example, "it may be easier to get a good answer if the doctor has sufficient time to phrase it." Others found it difficult to remember what the doctor said, for example, "the consultations are so rushed that it is hard to catch all that has been said," or that a written answer can be read more than once, thereby making it easier to understand.

## Discussion

In the present study we found that, of the multiple-choice alternatives, the reasons for consulting an Internet doctor in decreasing order were: convenience; anonymity; doctors too busy to answer questions; lack of time to visit a doctor; difficult to get an appointment; feeling uncomfortable when seeing a doctor; and financial reasons. In the free-text answers the reasons found were: second opinion; discontent with previous doctors; primary evaluation of a medical problem; convenience, distance and time; embarrassing concerns and worries; and preference for written communication. Half of the participants chose to give more than one reason for asking a doctor on the Internet. It is not relevant to directly compare the frequencies of the multiple-choice answers with the frequencies of the free-text answers, because the threshold for writing a free-text answer is higher than the threshold for choosing a multiple-choice alternative.

### Methods

The age profile of both the inquirers and the participants in the present study differs from the one seen in regular health care. The age of the majority of the inquirers of the Ask the Doctor service was 21-40 years. In spite of a low response rate the largest number of completed surveys also originated from this age group. Internet use has been found to be markedly age-related with the highest rates among youths and young adults [16- 17].

As men are regarded as more technology oriented than women, one might expect that men were more prone to use an Internet based Ask the Doctor service than women. However, during the period of the survey the use was dominated by women, with almost 3 out of 4 (71%) inquirers being women, thus exceeding the difference seen in regular Swedish health care. This gender difference corroborates other studies that have shown that women are more likely than men to go online to seek health-related information [18].

Internet users are a selected sample of the population. Sampling error (surveying a sample rather than the entire population) is a general dilemma in research and is a more pronounced problem in online research. The participants of the present Web survey chose to turn to the Internet with their medical issues. It is likely that they felt more positively about Internet based consultations than a population that has never considered the possibility of consulting an Internet doctor would. In line with this, the conclusions of the present survey should not be generalized to the population as a whole, but only to Ask the Doctor users who chose to participate in our survey. It is possible that the 66% of users who did not answer the survey had different reasons for consulting the service.

One of the advantages of Web surveys is that the effort required for gathering even large amounts of data is minimal. In our study 1223 surveys were completed. A disadvantage of Web surveys is the low response rate, in our survey 34%. Response rates in Web surveys are generally low, often far lower than in the present survey [19]. The shorter the survey is, the higher the response rate is likely to be. Trying to achieve an acceptable response rate in a Web survey while still being able to gather sufficient information is a question of balance. Our solution to this question was to combine quickly-entered multiple-choice answer options with an open-ended text box. As a result, the survey could be completed within a few minutes. A risk with this combination is that the multiple-choice alternatives presented before the free-text option could bias the free-text answer.

In the free-text responses we found some important information not included in the multiple-choice alternatives, thus the free-text option fulfilled its purpose.

The reliability of Web surveys compared to paper-and-pencil questionnaires can be disputed. In a comparative study of personality questionnaires performed either with paper-and-pencil or on the Internet there were no important differences to be found [20]. Another study compared patients' experiences of their physician's counseling using parallel telephone and Web surveys with exactly the same questions [21]. All the responses were uniform with the exception that the online participants were more overtly negative to previous counseling by their physicians than the telephone respondents were, suggesting that a spoken dialogue may restrain negative opinions. In the free-text responses of the present study we also found that a considerable number of the participants were overtly dissatisfied with previous performances of physicians. Thus, Web surveys could be an alternative to consider when it is important to get answers on sensitive issues such as an evaluation of the performance of a doctor.

### Results

Because computers are easily accessible, in homes as well as in workplaces, in most developed countries it is easy to understand why convenience was a major reason for participants choosing Internet consultations. Furthermore, the asynchronous access to the Internet based Ask the Doctor service allows users to access the service at times they find convenient, a feature appreciated by many of the participants.

In the Internet consultation the individual may remain anonymous thereby allowing inquirers to ask, eg, sensitive and embarrassing questions. In our study more than one third of the participants appreciated the opportunity of being able to ask anonymously, suggesting that this feature may supplement regular health care. In previous studies "health seekers" also appreciated the anonymity of searching the Internet for medical information [22,23].

A further reason given for using an Internet based Ask the Doctor service was, not surprisingly, the wish to be better informed. In spite of having previously visited a physician, many of the participants still had unfulfilled information demands, which corroborates earlier studies with similar results [1]. One fifth of the participants found doctors to be too busy to answer questions, a finding supported by many of the free-text answers. A frequent theme in the free-text answers was discontent with physicians. Thus, as noted before [1], Ask the Doctor services may act as an arena for the dissatisfied patient.

We found that many participants expressed a need for a second opinion, which may be one of the major features that Internet Ask the Doctor services can provide. In Sweden the right to a second opinion is granted only in the case of serious health conditions. For less-serious medical problems, or if there is a communication failure with the regular doctor, it is difficult to receive another doctor's evaluation of one's health problems.

The individual's preferred method of communication seems to be another important feature. Some participants stressed the importance of being able to reflect on both their question and the answer. Others responded that it could be difficult to understand what doctors said or that the information could be hard to remember. The complexity of today's medical situations, where there are several treatment options, could be a reason to provide more of the information in writing.

The low importance in our study of financial reasons for consulting Internet doctors is probably due to the relatively low cost of medical care for the individual in Sweden. In contrast in a study including many international inquirers 14% of the participants claimed that they could not afford local medical services [13] compared to 3% in our study of, presumably, mainly Swedish citizens.

### Limitations and Risks

A crucial issue is whether consultations on the Internet might cause harm for the involved individuals or their regular doctors. Until now there have been only a few reports on harm related to the use of the Internet [24- 25]. MedCERTAIN (now MedCIRCLE), a international collaboration of trusted organizations active in the field of rating and annotating health information, has set up a database, DAERI (Database of Adverse Events Related to the Internet) to collect such reports [26,27]. One aspect of possible harm is the risk of negative impact on the relationship between the inquirer and the inquirer's regular doctor. An insensitive answer from an Internet doctor might reduce the inquirer's confidence in the regular doctor. In a British survey, twice as many doctors reported patients experiencing benefits than problems from the Internet [28].

It has been claimed by doctors that it is preferable for all patients searching for medical advice to see a doctor in a face-to-face consultation [29]. However, in some situations individuals may instead choose to consult an Internet doctor. Some patients also want to communicate with their doctors by e-mail [30- 33].

### The Future

We can probably anticipate an increasing demand for Internet based and e-mail consultations as a component in the patients' mix of communicating with doctors, even if these services will be subject to fees [31]. The development of more sophisticated technologies including image and sound may partly overcome some of the limitations implied by the lack of a physical examination.

It is probable that different types of Internet based services will be regular parts of the services offered by most health care providers. Such a development will require ethical guidelines [9], education, and training, as well as standards concerning communication records. With regard to Internet based consultations between individuals and doctors, further studies are required to answer a number of questions: In what way do these inquirers differ from those visiting regular health care centers? Will the inquirers raise other kinds of complaints? How should health personnel, in particular doctors, adapt to this new situation?

### Conclusions

We found that an Internet based Ask the Doctor service is primarily consulted because it is convenient, but that it may also be of value for individuals with needs that regular health care services have not been able to meet. The Internet based Ask the Doctor service we studied provided an arena for sensitive questions, for individuals seeking advice on behalf of relatives, and for inquirers preferring written communication. In spite of the limitations implied by their lack of a personal meeting and a physical examination, Internet based Ask the Doctor services are of value for individuals with needs that regular health care services have not been able to fully satisfy.

## References

[1] Eysenbach G, Diepgen T L (1999). Patients looking for information on the Internet and seeking teleadvice: motivation, expectations, and misconceptions as expressed in e-mails sent to physicians. Arch Dermatol.

[2] Borowitz S M, Wyatt J C (1998). The origin, content, and workload of e-mail consultations. JAMA.

[3] Spielberg A R (1998). On call and online: sociohistorical, legal, and ethical implications of e-mail for the patient-physician relationship. JAMA.

[4] Ferguson T (1998). Digital doctoring--opportunities and challenges in electronic patient-physician communication. JAMA.

[5] Eysenbach G, Diepgen T L (1998). Responses to unsolicited patient e-mail requests for medical advice on the World Wide Web. JAMA.

[6] Kane B, Sands DZ (1998). Guidelines for the clinical use of electronic mail with patients. The AMIA Internet Working Group, Task Force on Guidelines for the Use of Clinic-Patient Electronic Mail. J Am Med Inform Assoc.

[7] Huntley A C (1999). The need to know: patients, e-mail, and the Internet. Arch Dermatol.

[8] Lewis A D (2000). Patients, physicians, and e-mail. Arch Dermatol.

[9] Eysenbach G (2000). Towards ethical guidelines for dealing with unsolicited patient emails and giving teleadvice in the absence of a pre-existing patient-physician relationship   systematic review and expert survey. J Med Internet Res.

[10] Deville K, Fitzpatrick J (2000). Ready or not, here it comes: the legal, ethical, and clinical implications of E-mail communications. Semin Pediatr Surg.

[11] Kuszler P C (2000). A question of duty: common law legal issues resulting from physician response to unsolicited patient email inquiries. J Med Internet Res.

[12] Sittig D F, King S, Hazlehurst B L (2001). A survey of patient-provider e-mail communication: what do patients think?. Int J Med Inform.

[13] Labiris G, Coertzen I, Katsikas A, Karydis A, Petounis A (2002). An eight-year study of internet-based remote medical counselling. J Telemed Telecare.

[14] Infomedica Home page.

[15] Strauss Anselm, Corbin Juliet M (1990). Basics of Qualitative Research : Grounded Theory Procedures and Techniques.

[16] US Department of Commerce (2002). A nation online: how Americans are expanding their use of the Internet. US Department of Commerce, Economics and Statistics Administration.

[17] Coleman N, Jeawody F, Wapshot J Electronic government at Department for Work and Pensions: attitudes to electronic methods of conducting benefit. Department for Work and Pensions Research Report No. 176.

[18] Fox S, Fallows D (2003). Internet health resources: health searches and email have become more commonplace, but there is room for improvement in searches and overall Internet access.

[19] Vehovar V, Lozar K, Batagelj Z, Zaletel M, Groves Robert M, Dillman Don A, Eltinge John L, Little RJA (2001). Nonresponse in Web surveys. Survey Nonresponse (Wiley Series in Survey Methodology).

[20] Pettit Frances Annie (2002). A comparison of World-Wide Web and paper-and-pencil personality questionnaires. Behav Res Methods Instrum Comput.

[21] Taylor H (2000). Does Internet research work?. International journal of market research.

[22] Fox Susannah (2000). The online health care revolution: How the web helps Americans take better care of themselves.

[23] Rideout V (2001). Generation Rx.com: how young people use the Internet for health information.

[24] Smith R (2001). Almost no evidence exists that the internet harms health. BMJ.

[25] Crocco Anthony G, Villasis-keever Miguel, Jadad Alejandro R (2002). Analysis of cases of harm associated with use of health information on the internet. JAMA.

[26] Eysenbach Gunther, Köhler Christian (2002). Does the internet harm health? Database of adverse events related to the internet has been set up. BMJ.

[27] Research Unit for Cybermedicine & E-health Database of Adverse Events Related to the Internet (DAERI).

[28] Potts HWW, Wyatt JC (2002). Survey of doctors' experience of patients using the Internet. J Med Internet Res.

[29] Standing Committee of European Doctors (CP) (1997). Ethical guidelines in telemedicine.

[30] Couchman G R, Forjuoh S N, Rascoe T G (2001). E-mail communications in family practice: what do patients expect?. J Fam Pract.

[31] Harris Interactive (2002). Patient/physician online communication: many patients want it, would pay for it, and it would influence their choice of doctors and health plans. Harris Interactive Health Care News.

[32] Andreassen Hege, Sandaune Anne-Grete, Gammon Deede, Hjortdahl Per (2002). [Norwegian use of Internet health services]. Tidsskr Nor Laegeforen.

[33] Poensgen A, Larsson S (2001). Patients, physicians, and the Internet: myth, reality, and implications.

